# Mechanisms of Compound Kushen Injection for the Treatment of Lung Cancer Based on Network Pharmacology

**DOI:** 10.1155/2019/4637839

**Published:** 2019-05-28

**Authors:** Ziqi Meng, Xinkui Liu, Jiarui Wu, Wei Zhou, Kaihuan Wang, Zhiwei Jing, Shuyu Liu, Mengwei Ni, Xiaomeng Zhang

**Affiliations:** ^1^Department of Clinical Chinese Pharmacy, School of Chinese Materia Medica, Beijing University of Chinese Medicine, Beijing 100102, China; ^2^Department of Academic Management, China Academy of Chinese Medicine Sciences, Beijing 100700, China

## Abstract

**Background:**

Compound Kushen Injection (CKI) is a Chinese patent drug that shows good efficacy in treating lung cancer (LC). However, its underlying mechanisms need to be further clarified.

**Methods:**

In this study, we adopted a network pharmacology method to gather compounds, predict targets, construct networks, and analyze biological functions and pathways. Moreover, molecular docking simulation was employed to assess the binding potential of selected target-compound pairs.

**Results:**

Four networks were established, including the compound-putative target network, protein-protein interaction (PPI) network of LC targets, compound-LC target network, and herb-compound-target-pathway network. Network analysis showed that 8 targets (CHRNA3, DRD2, PRKCA, CDK1, CDK2, CHRNA5, MMP1, and MMP9) may be the therapeutic targets of CKI in LC. In addition, molecular docking simulation indicated that CHRNA3, DRD2, PRKCA, CDK1, CDK2, MMP1, and MMP9 had good binding activity with the corresponding compounds. Furthermore, enrichment analysis indicated that CKI might exert a therapeutic role in LC by regulating some important pathways, namely, pathways in cancer, proteoglycans in cancer, PI3K-Akt signaling pathway, non-small-cell lung cancer, and small cell lung cancer.

**Conclusions:**

This study validated and predicted the mechanism of CKI in treating LC. Additionally, this study provides a good foundation for further experimental studies and promotes the reasonable application of CKI in the clinical treatment of LC.

## 1. Introduction

Lung cancer (LC) has become an important etiology of malignant mortality worldwide [1]. Approximately 1.37 million deaths annually worldwide have been attributed to LC, and LC is a significant cause of cancer-related deaths in China [2, 3]. LC is divided into several different categories, such as lung adenocarcinoma, squamous cell lung carcinoma, large cell carcinoma and small cell lung carcinoma (SCLC) [4]. Current treatment strategies for LC include surgery, chemotherapy, radiation therapy, laser therapy, and photodynamic therapy [5–7]. The most important therapeutic tactic for LC is chemotherapy, but invasion and metastasis of LC cells often occur after chemotherapy [8].

Because traditional Chinese medicine (TCM) can alleviate uncomfortable symptoms, improve survival benefits, and reduce the side effects of chemotherapy, TCM has become one of the crucial options for comprehensive cancer treatments [9, 10]. Compound Kushen Injection (CKI, also known as Yanshu injection) is a TCM preparation derived from two herbs, kushen (*Radix Sophorae Flavescentis*), and baituling (*Rhizoma Smilacis Glabrae*) [11, 12]. Matrine and oxymatrine are the primary active compounds in CKI and have antitumor effects in different cancer cells, including breast cancer cell lines (MCF-7), gastric cancer cells (SGC-7901 and MKN45), and human liver cancer cells (SMMC-7721) [13, 14]. Many clinical studies have confirmed that CKI can be used to treat malignant tumors by inducing apoptosis in tumor cells, as well as by inhibiting cancer cell proliferation and tumor growth, migration and invasion [13, 15, 16]. Moreover, a recent study has systematically investigated the mechanism of CKI in treating hepatocellular carcinoma based on a network pharmacology method [17]. Additionally, CKI also synergizes the curative effects of radiotherapy and chemotherapy for cancer patients as well as mitigates radiotherapy and chemotherapy toxic side effects [18, 19]. In particular, CKI relieves the pain of cancer patients, which is of great significance to improve their quality of life and prolong their survival [20, 21].

TCM serves as a multicomponent, multitarget, and multipathway therapy that acquires its special treatment efficacy by acting on the biological network of body systems, and thereby, it is difficult to elucidate the mechanisms of TCM [22, 23]. Fortunately, network pharmacology provides a new perspective for promoting a new understanding of the mechanisms of drugs [24, 25]. Network pharmacology updates the “one target, one drug” model to the “multicomponent, multitarget” model and better elucidates the complex interactions among genes, proteins, and metabolites associated with diseases and drugs from a network perspective [26, 27].

**Figure 1:**
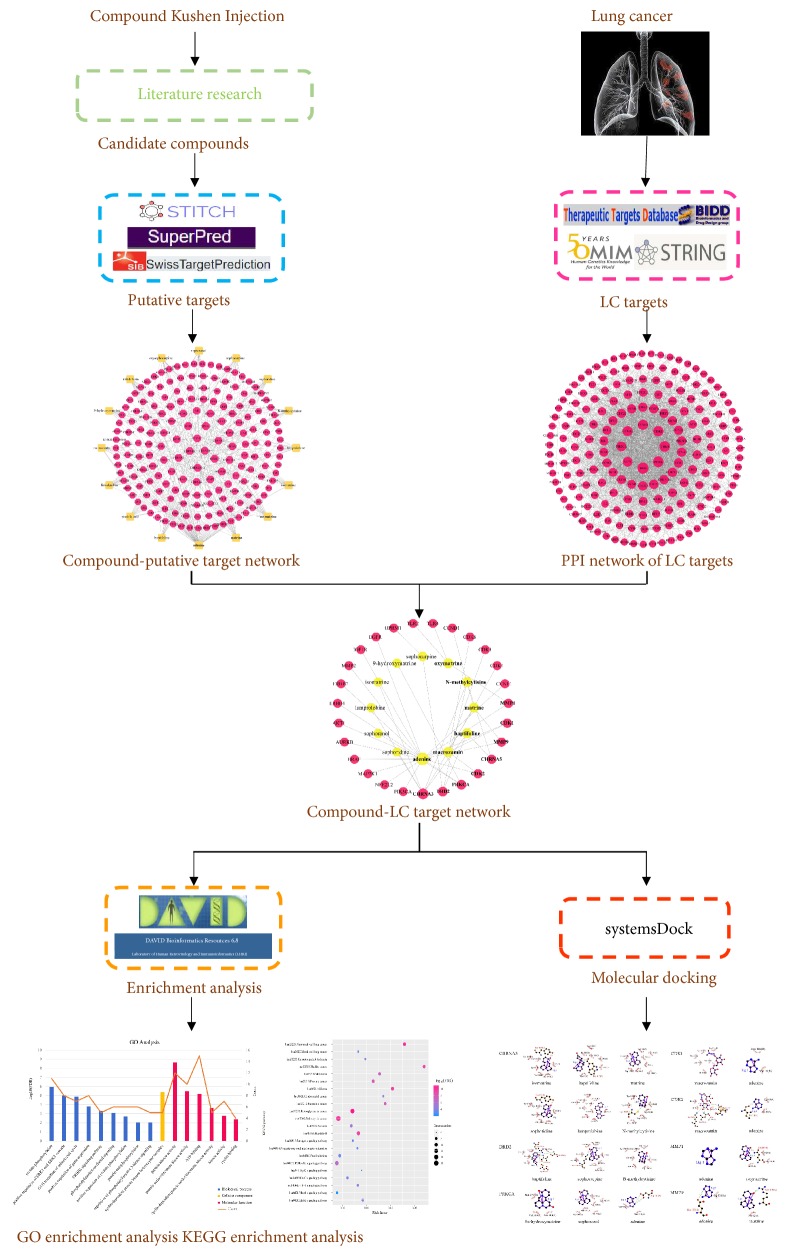
Flowchart for CKI in treating LC.

Although several studies have indicated that CKI has efficacy in the treatment of LC [28–30], its molecular mechanisms have not been completely elucidated. Therefore, our study adopted the network pharmacology method to better investigate and predict the molecular mechanism of CKI against LC. A detailed flowchart of the network pharmacology-based study is shown in Figure 1.

## 2. Materials and Methods

### 2.1. Chemical Components of CKI

After searching the literature [31, 32], 23 compounds of CKI were selected to further study. We inputted all the compounds into the PubChem database [33] (https://pubchem.ncbi.nlm.nih.gov), and then removed the compounds with duplicated data and without structural information. In total, 16 chemical components were collected and these compounds were clustered by K-means clustering algorithm based on their molecular descriptors.

### 2.2. Putative Targets of CKI

The simplified molecular input entry specification (SMILES) information of 16 compounds was imported into the Search Tool for Interacting Chemicals (STITCH), SuperPred, and SwissTargetPrediction databases. STITCH [34] (http://stitch.embl.de/) is a database of known and predicted interactions between compounds and proteins, and the database is based on text mining and molecular docking techniques to predict interactions between compounds and proteins. STITCH has been used to study TCM to find the potential active components and to explain the molecular mechanism of TCM [35]. The SuperPred database [36] (http://prediction.charite.de/) is based on the principle of similarity that links the chemical similarity of drug-like compounds to molecular targets and the therapeutic approach. SwissTargetPrediction [37] (http://www.swisstargetprediction.ch/) is a web server based on chemical similarity that can accurately predict bioactive molecular targets. Finally, we obtained corresponding targets after discarding duplicate data.

### 2.3. LC Targets

Different genes related to LC were gathered from the Therapeutic Target Database (TTD, https://db.idrblab.org/ttd/), which is a database that provides information about nucleic acid targets and therapeutic effects of proteins [38], and the Online Mendelian Inheritance in Man [39] (OMIM, http://www.omim.org/), which is an online database of continuously updated human genes and genetic diseases. The keyword “lung cancer” was used in the TTD and OMIM databases to search for LC-related targets.

### 2.4. Protein-Protein Interaction Data

Protein-protein interaction (PPI) data were extracted from Search Tool for the Retrieval of Interacting Genes/Proteins (STRING, https://string-db.org/). STRING is a database of known and predicted PPIs, including both direct and indirect interactions among proteins [40]. STRING defines PPIs with confidence ranges for data scores (low: <0.4; medium: 0.4 to 0.7; high: >0.7). We inputted the LC-related targets into the STRING database, with the species limited to “*Homo sapiens*” and the confidence scores higher than 0.7.

### 2.5. Network Construction

We constructed four networks in this study as follows: (1) a compound-putative target network was established by linking chemical compounds of CKI and corresponding targets; (2) the PPI network of LC targets was built by connecting LC-related targets and other human proteins that linked or interacted with LC targets; (3) a compound-LC target network was constructed by intersecting the compound-putative target network and the PPI network of LC targets. The genes that did not intersect were removed; namely, the common targets between the compound-putative target network and the PPI network of LC targets were the potential targets for the ingredients of CKI in LC; and (4) an herb-compound-target-pathway network was built by linking herbs, compounds, corresponding targets, and pathways.

Cytoscape 3.5.1 software [41] (http://www.cytoscape.org/) was used to construct all of the above networks. Cytoscape is bioinformatics analysis software that is applied to visual biological pathways and intermolecular interaction networks. Cytoscape provides a set of basic data integration, analysis, and visualization functions to analyze complex networks. For each node in the interaction network, a significant parameter “Degree” is calculated to evaluate its topological features. A degree is defined as the number of edges to node* i* [41, 42]. The higher the degree is, the more important the node is.

### 2.6. Molecular Docking Simulation

SystemsDock [43] (http://systemsdock.unit.oist.jp/iddp/home/index) is an emerging web server for network pharmacology-based prediction and analysis and illustrates the mechanism of ligand acting on a complex molecular network by applying high-precision docking simulation and molecular pathway map. The docking score of systemsDock is a negative logarithm of the experimental dissociation/inhibition constant (pKd/pKi), which can directly indicate the binding strength [44]. We used systemsDock to evaluate the binding potential between selected targets and corresponding compounds in the compound-LC target network.

### 2.7. Gene Ontology and Pathway Enrichment

To illustrate the role of potential targets in gene function and signal pathways, we used the Database for Annotation, Visualization and Integrated Discovery [45] (DAVID, https://david.ncifcrf.gov/) to perform Gene Ontology (GO) function enrichment analysis and Kyoto Encyclopedia of Genes and Genomes (KEGG) pathway enrichment analysis of genes in the compound-LC target network.

## 3. Results

### 3.1. Compound-Putative Target Network

**Figure 2:**
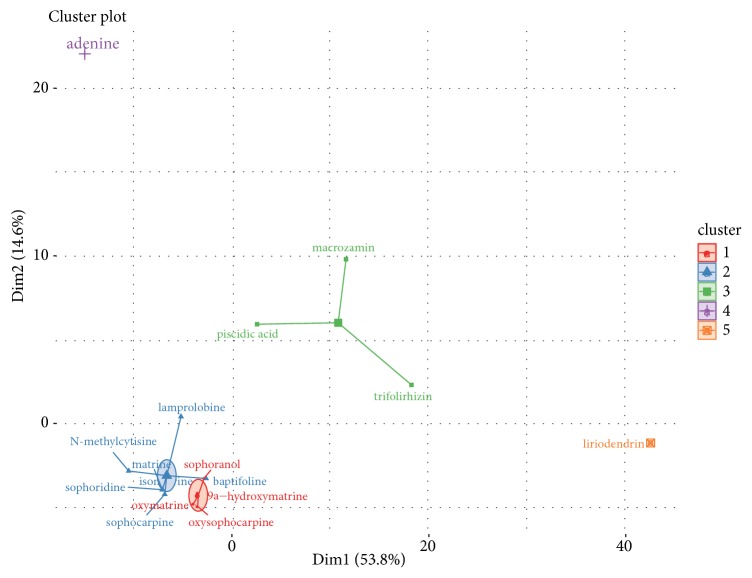
Cluster plot of 16 compounds.

**Figure 3:**
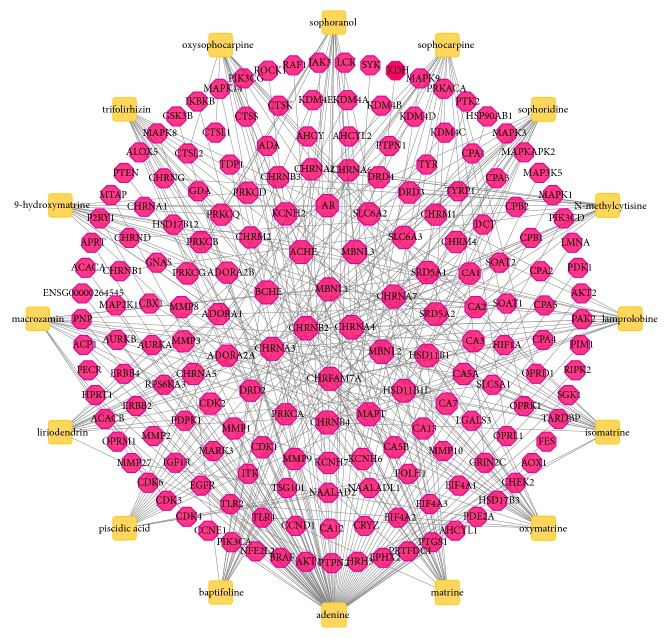
Compound-putative target network of CKI. Yellow rectangles represent compounds in CKI. Red octagons represent corresponding targets. There is a positive proportional relationship between the node size and the degree.

The details of the 16 compounds in CKI are described in Supplementary Table S1. And these compounds were clustered into 5 clusters (Figure 2). As shown in Figure 3, the compound-putative target network included 196 nodes (16 compound nodes and 180 putative target nodes) and 326 edges. In this network, many putative targets were regulated by multiple compounds. For example, CHRNA4 and CHRNB2 were modulated by matrine, oxymatrine, and so on. In addition, network analysis showed that the average degree value of compounds was 20.38, indicating the properties of multitargets of CKI in treating LC. Notably, there were 3 compounds with degree ≥ 20.38, namely, adenine (degree = 72), matrine (degree = 21), and oxymatrine (degree = 21), demonstrating their significance in the network.

### 3.2. PPI Network of LC Targets

**Figure 4:**
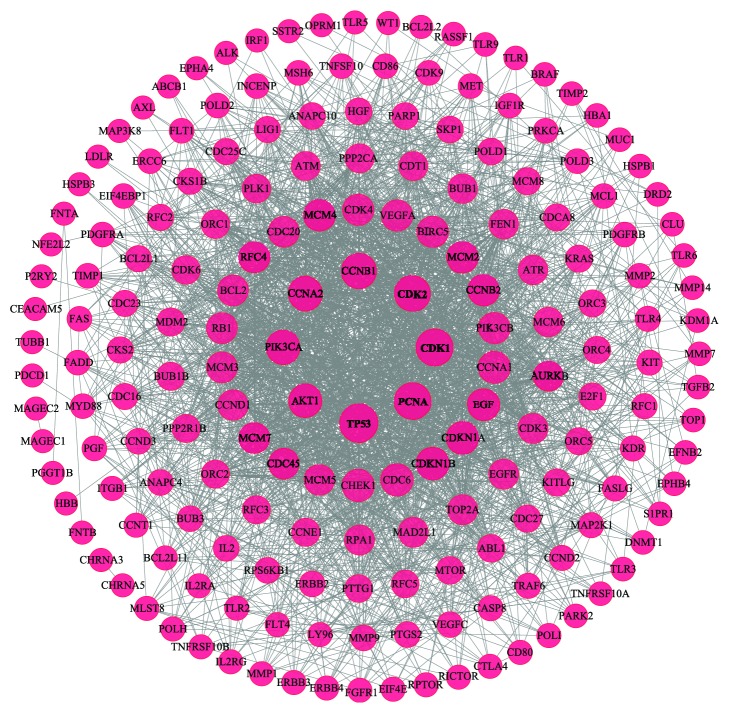
PPI network related to LC. There is a positive proportional relationship between the node size and the degree.

A total of 97 LC targets were retrieved from the TTD and OMIM databases (as shown in Supplementary Table S2). In Figure 4, the PPI network of LC targets was composed of 188 LC-related genes and 2019 LC-associated PPIs. Three topological features of each node in the network were calculated to find the major nodes. Ultimately, 26 nodes with an average degree value ≥ 21.48, betweenness ≥ 0.01251 and closeness ≥ 0.4547 were selected as major nodes (the details about the information of 26 nodes are described in Supplementary Table S3). Therefore, these genes might be the key genes in the development of LC.

### 3.3. Compound-LC Target Network

**Figure 5:**
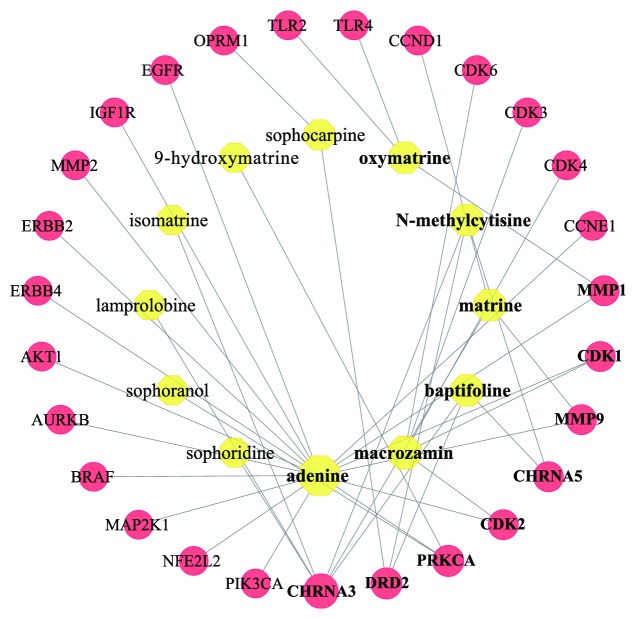
Compound-LC target network. Yellow octagons represent compounds in CKI. Red circles represent potential targets for CKI in the treatment of LC. There is a positive proportional relationship between the node size and the degree.

The compound-LC target network is shown in Figure 5 and includes 39 nodes (12 compound nodes and 27 target nodes) and 41 edges. The network showed that the 27 notes might serve as potential targets of CKI for treating LC (Supplementary Table S4). To find the major nodes, we selected 8 targets based on an average value of degree ≥ 1.52, namely, neuronal acetylcholine receptor subunit alpha-3 (CHRNA3), D(2) dopamine receptor (DRD2), protein kinase C alpha type (PRKCA), cyclin-dependent kinase 1 (CDK1), cyclin-dependent kinase 2 (CDK2), neuronal acetylcholine receptor subunit alpha-5 (CHRNA5), interstitial collagenase (MMP1), and matrix metalloproteinase-9 (MMP9), indicating that they were likely to be potential targets of CKI for the treatment of LC. In addition, 6 compounds were selected with degree ≥ 3, such as adenine, macrozamin, matrine, oxymatrine, baptifoline, and N-methylcytisine, illustrating that these compounds were likely to be key compounds of CKI in treatment for LC.

### 3.4. Molecular Docking Verification

**Figure 6:**
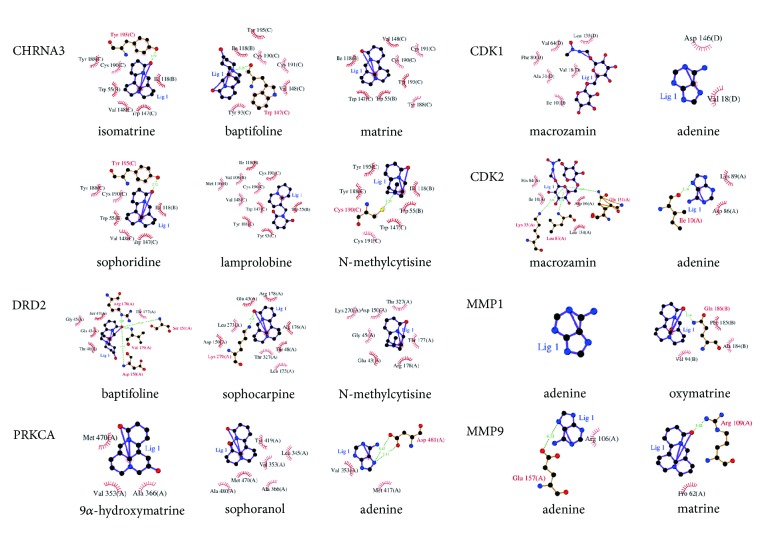
The detailed target-compound interactions of the docking simulation

The 3D structures of the above 8 selected targets were gathered from the PDB database [46] (https://www.rcsb.org/), which is the single global archive and includes experimentally determined atomic-level 3D structures of biological macromolecules (proteins, DNA, and RNA). The results showed that 7 targets (CHRNA3, DRD2, PRKCA, CDK1, CDK2, MMP1, and MMP9) had 3D structures, while the 3D structure of CHRNA5 was not available. These 7 targets were inputted into systemsDock for molecular docking verification. As shown in Table 1, a total of 20 pairs of target-compound combinations were delivered into docking. The docking scores of most of them were larger than 5.52, which showed that they possessed good binding activity [47]. The details of the target-compound interactions of the docking simulation are shown in Figure 6.

### 3.5. GO and Pathway Enrichment Analysis

**Figure 7:**
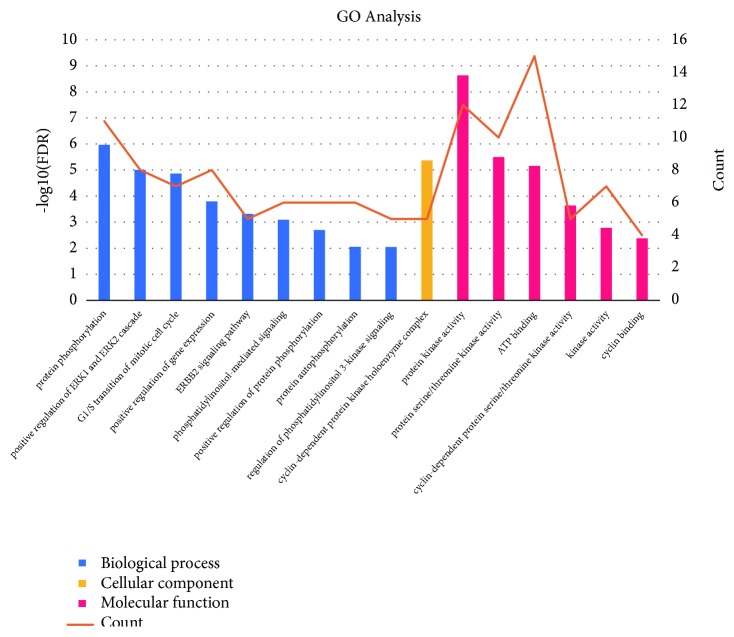
GO analysis of potential targets. The* Y*-axis shows the enrichment scores of these terms or the counts of targets, and the* X*-axis shows significantly enriched GO categories of the target genes (FDR < 0.01).

To elucidate the multiple mechanisms of CKI on LC from a systematic level, we performed GO enrichment analysis for 27 targets of the compound-LC target network. In total, 16 enriched GO terms were identified (FDR < 0.01, as shown in Figure 7). For biological processes, the targets of CKI were enriched in protein phosphorylation (GO:0006468), positive regulation of ERK1 and ERK2 cascade (GO:0070374), G1/S transition of mitotic cell cycle (GO:0000082), positive regulation of gene expression (GO:0010628), ERBB2 signaling pathway (GO:0038128), phosphatidylinositol-mediated signaling (GO:0048015), positive regulation of protein phosphorylation (GO:0001934), protein autophosphorylation (GO:0046777), and regulation of phosphatidylinositol 3-kinase signaling (GO:0014066). Protein kinase activity (GO:0004672), protein serine/threonine kinase activity (GO:0004674), ATP binding (0005524), cyclin-dependent protein serine/threonine kinase activity (GO:0004693), kinase activity (GO:0016301), and cyclin binding (GO:0030332) were the particularly enriched molecular functions. For cellular components, the targets of CKI were enriched in cyclin-dependent protein kinase holoenzyme complex (GO:0000307).

**Figure 8:**
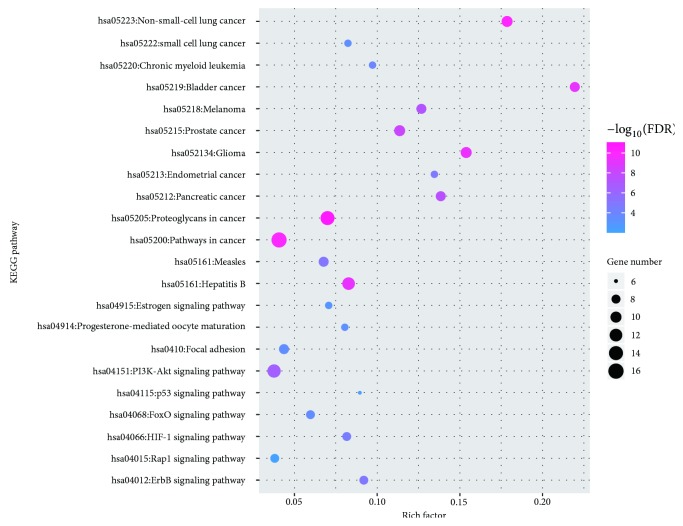
KEGG analysis of potential targets. The* Y*-axis shows significantly enriched KEGG pathways of the target genes, and the* X*-axis shows the rich factor (FDR < 0.01). The rich factor represents the ratio of the number of target genes belonging to a pathway to the number of all the annotated genes located in the pathway. A higher rich factor represents a higher level of enrichment. The size of the dot indicates the number of target genes in the pathway, and the color of the dot reflects the different *P* value range.

**Figure 9:**
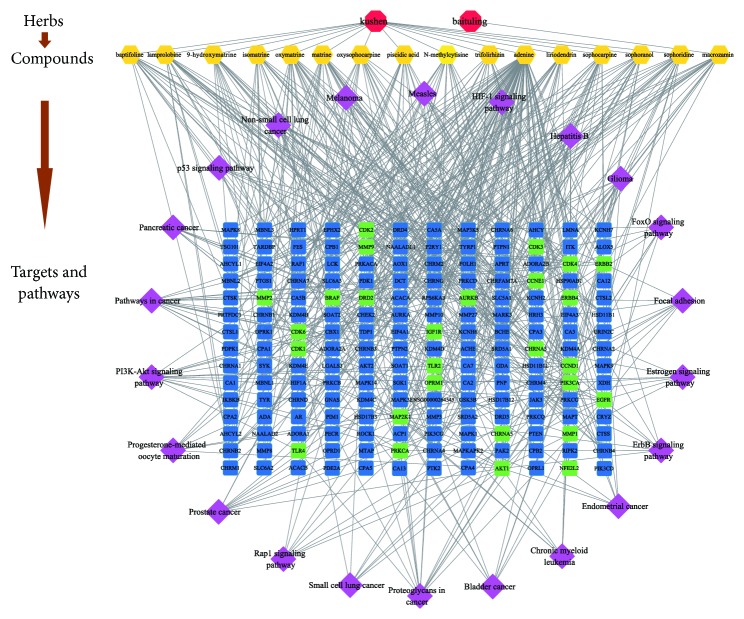
Herb-compound-target-pathway network. Red octagons represent the 2 herbs in CKI. Yellow hexagons represent 16 compounds of 2 herbs. Blue rectangles represent corresponding targets. Green rectangles represent 27 potential targets of CKI in the treatment of LC. Purple diamonds represent 22 corresponding pathways involving 27 potential targets.

To illustrate the crucial pathways among the 27 potential targets in LC treatment, we screened 22 pathways according to FDR < 0.01 (as shown in Figure 8), including proteoglycans in cancer (hsa05205), non-small-cell lung cancer (hsa05223), pathways in cancer (hsa05200), glioma (hsa05214), bladder cancer (hsa05219), prostate cancer (hsa05215), pancreatic cancer (hsa05212), melanoma (hsa05218), endometrial cancer (hsa05213), chronic myeloid leukemia (hsa05220), small cell lung cancer (hsa05222), FoxO signaling pathway (hsa04068), focal adhesion (hsa04510), progesterone-mediated oocyte maturation (hsa04914), estrogen signaling pathway (hsa04915), p53 signaling pathway (hsa04115), rap1 signaling pathway (hsa04015), hepatitis B (hsa05161), PI3K-Akt signaling pathway (hsa04151), measles (hsa05162), ErbB signaling pathway (hsa04012), and HIF-1 signaling pathway (hsa04066). Finally, based on the above information, we constructed an herb-compound-target-pathway network (Figure 9) to holistically explain the mechanism of CKI in treating LC.

## 4. Discussion

CKI has been clinically used to treat various types of solid tumors [48]. Currently, CKI has been widely used for pain treatment in combination with chemotherapy and radiotherapy throughout China [49]. In the present work, we constructed a compound-putative target network, PPI network of LC targets, compound-LC target network, and herb-compound-target-pathway network to systematically analyze the mechanism of CKI in the treatment of LC.

In the compound-putative target network, adenine, matrine, and oxymatrine were recognized as significant compounds. Adenine was shown to be associated with a variety of LC targets, including CDK1, CDK2, MMP1, MMP9, and so on. Matrine is widely known as the main chemical composition of CKI and matrine derivatives containing the benzo-*α*-pyrone structure are potent antilung cancer agents [50]. A recent study indicated that matrine inhibits the migration and invasion of non-small-cell lung cancer (NSCLC) cells by interfering with the epithelial-mesenchymal transition signaling pathway [51]. Moreover, the matrine derivative YF-18 inhibits the growth and migration of LC cells by inducing G2/M cell cycle arrest and downregulating Skp2 [52]. The present research found that matrine was associated with some LC-related targets, including MMP9, CHRNA3, and CCND1. Moreover, previous studies have also demonstrated that matrine could decrease the expression of MMP9 and cyclin D1 [53, 54]. For oxymatrine, modern studies have demonstrated that oxymatrine has anticancer potential in various types of cancer cells by multiple mechanisms [55–57]. Furthermore, a relevant report implied that oxymatrine inhibits NSCLC by suppressing the activity of the EGFR signaling pathway [58]. In addition, oxymatrine has been proven to be a restrainer of TLR2 and TLR4 and an agonist of MMP1 [59, 60]. Fortunately, our current study also discovered that oxymatrine interacted with the above three LC-related targets. However, some results of our study have been rarely reported, and thus, further studies are urgently needed to validate our results.

In the PPI network of LC targets, TP53, PCNA, CDK1, and CDK2, which possessed high values for degree, betweenness, and closeness, were selected as major nodes. TP53 is a significant tumor suppressor gene that is closely related to the cell cycle, proliferation, differentiation, aging, and apoptosis [61]. Meanwhile, gene alteration of TP53 is associated with a poor prognosis in patients with NSCLC [62]. PCNA is a key eukaryotic replication accessory factor that plays an important role in the cell cycle and apoptosis [63]. In addition, PCNA can promote LC progression [64]. CDK1 and CDK2 belong to the cyclin-dependent kinases (CDKs) family. CDKs are a specific family of enzymes that turn on cell cycle mechanisms. CDKs require association with regulatory proteins (cyclins), and the absence of regulatory proteins at cell cycle checkpoints leads to carcinogenesis or tumor development [65]. The CDK2 and cyclin A complex plays a vital role in the G1 phase arrest of cancer cells [66]. A previous study has shown that the CDK2 inhibitor could elicit antineoplastic effects in LC [67]. The CDK1 gene plays an important role in the progression of cells from the G2 phase to the M phase. In addition, high expression of CDK1 is associated with poor prognosis in patients with advanced NSCLC [68]. Moreover, a study found that CDK1 was a direct target of miR-181a and that miR-181a inhibited cell proliferation by modulating CDK1 mRNA and protein levels in NSCLC cells [69]. Fortunately, our work found that CDK1 and CDK2 interact with multiple ingredients for CKI, including adenine, and macrozamin, and CKI has been shown to have therapeutic effects on cancer through inhibiting CDK2 activity [70]. In addition, matrine, the main active compound of CKI has been found to exhibit time-dependent inhibition of the expression of CDK1 in prostate cancer cells [71]. Hence, CKI might treat LC by modulating the expression of CDK1 and CDK2.

The compound-LC target network included 27 targets, and these targets mainly act on adenine, macrozamin, matrine, oxymatrine, baptifoline, and N-methylcytisine. The 8 targets with a degree ≥ 1.52 were determined as key targets, including CHRNA3, DRD2, PRKCA, CDK1, CDK2, CHRNA5, MMP1, and MMP9. Moreover, we inputted the above targets and corresponding compounds into systemsDock for molecular docking verification. The docking scores showed that most of them had good binding activity, especially the CHRNA3-isomatrine pair (6.802), CHRNA3-baptifoline pair (6.787), CHRNA3-matrine pair (6.783), CHRNA3-sophoridine pair (6.731), and PRKCA-9*α*-hydroxymatrine pair (6.657). For CHRNA3 and CHRNA5, several genome-wide association studies (GWAS) have ascertained that CHRNA3 and CHRNA5 are associated with the risk of LC [72]. CHRNA3 may be a more important candidate susceptibility gene for LC. A study has shown that the CHRNA3 subunit binds to nicotine-derived nitrosamine ketone (NNK), subsequently upregulating nuclear factor-kappa B (NF-*κ*B) to induce cell proliferation and increase the risk of LC [73]. For CHRNA5, a study has shown that knockdown of *α*5-nAChR, which is encoded by the CHRNA5 gene, could significantly mediate crux pathways, including cell cycle distribution, apoptosis, DNA replication and pathways in cancer. Additionally, silencing *α*5-nAChR restrains the progression of nicotine-related NSCLC [74]. Therefore, CKI might produce therapeutic effects by inhibiting the expression of CHRNA3 and CHRNA5. DRD2 was targeted by 3 compounds from CKI, including baptifoline, N-methylcytisine and sophocarpine. DRD2 is expressed in different pulmonary carcinoma cells [75]. Moreover, the DRD2 agonist quinpirole could counteract SCLC cell proliferation in a dose- and time-dependent manner [76]. Campa D et al. [77] indicated that DRD2 polymorphisms were related to a 2- to 5-fold increased risk of NSCLC. Thus, we deduced that the mechanism for treating LC might be associated with the activation of DRD2 by CKI. PRKCA is a serine-threonine kinase that is associated with various cellular functions [78]. Previous studies have proven that the PRKCA gene might have multiple effects on the lung, such as peribronchiolar cell proliferation and proinflammatory and profibrotic cytokine expression [79]. In addition, a study has shown that PKC*α* (PRKCA) is a significant protein in LC [80]. Other research has also demonstrated that PRKCA is a potential hub in LC-related signals [81]. Therefore, the mechanism of CKI for the treatment of LC may be related to its regulation of PRKCA expression. MMP1 and MMP9 are matrix metalloproteinase (MMP) genes that belong to a large family of zinc-dependent endopeptidases [82, 83]. The upregulation of MMP has been shown in various types of solid cancers [84]. A study has shown that the imbalance between MMPs and their depressors plays a significant role in the development of head and neck cancer and the prognosis of patients [85]. The expression of MMP1 is related to many diseases, including emphysema and malignant tumors [86]. A previous study demonstrated that the polymorphism MMP1 -1607 1G > 2G was significantly related to a remarkable increase in cancer risk [87]. A study by Yu et al. [88] found that the expression of MMP1 increased in the process of invasion and metastasis of NSCLC. Notably, our study showed that MMP1 was targeted by adenine and oxymatrine, and previous evidence has indicated that oxymatrine inhibits the proliferation and facilitates apoptosis of cancer cells through downregulating MMP2 and MMP9 expression [89–91]. However, the relationship among oxymatrine, MMP1, and LC cells is still unclear, which needs to be further explored. MMP9 is undetectable in healthy tissue, but during inflammation and cancer it is highly upregulated [92]. A study has indicated that MMP9 is regulated by myocardial infarction-associated transcript (MIAT) to affect the migration and invasion of NSCLC [93]. Furthermore, an existing study has found that 3T3-L1 adipocyte-derived exosomes promote murine 3LL Lewis LC cells invasion* in vitro* through increasing MMP9 activity [94]. The present study found that MMP9 was targeted by adenine and matrine, and several studies have shown that matrine could reduce the level of MMP9 in cancer cells [53, 95]. In addition, CKI has been proved to have the suppression action for growth and migration of LC cells by inhibiting MMP9 expression [96]. Therefore, this indicated that CKI produced the healing efficacy for LC by regulating MMP1 and MMP9 expression.

In this study, we performed a GO enrichment analysis to clarify the multiple mechanisms of CKI against LC from a systematic level. These targets were highly associated with G1/S transition of mitotic cell cycle, ERK1 and ERK2 cascade, protein phosphorylation, protein kinase activity, and ATP binding in the GO enrichment analysis. Thus, the results demonstrated that CKI mainly produces therapeutic effects by participating in these biological processes and molecular functions.

In the pathway enrichment, we found that the pathways directly associated with LC were non-small-cell lung cancer (hsa05223) and small cell lung cancer (hsa05222). Histological subtypes of LC mainly included NSCLC and SCLC; approximately 85% of all LC cases are NSCLC, and 15% are SCLC [97, 98]. In addition, pathways in cancer (hsa05200) were identified as a significant pathway in LC treatment with the highest number of genes. In this pathway, the genes in the compound-LC target network were members of many vital subpathways, such as the PI3K-Akt signaling pathway The other significantly enriched pathway was proteoglycans in cancer (hsa05205), and it also included the PI3K-Akt signaling pathway as an important subpathway. The PI3K-Akt signaling pathway (hsa04151) regulates numerous cellular functions, such as cell growth, proliferation, differentiation, survival, and invasion, and modulates the occurrence of NSCLC [99]. Moreover, the PI3K-Akt pathway is involved in cell apoptosis in multiple cell lines, such as LC cell lines [100]. A study has demonstrated that MMP9 promotes the development of LC through the PI3K-Akt signaling pathway [101]. Other research has shown that matrine regulates the expression of related genes and proteins through the PI3K-Akt signal transduction pathway to induce apoptosis in A549 cells, a LC cell line [102]. Therefore, it was speculated that the ingredients of CKI might play a significant role in treating LC through key factors in these signaling pathways.

## 5. Conclusions

In the present study, we found 16 compounds of CKI and predicted 180 putative targets, proving that CKI was a complex preparation with the multicomponent and multitarget features. CKI exerted treatment effects on LC by regulating 27 targets, which were mainly connected to adenine, macrozamin, matrine, oxymatrine, baptifoline, and N-methylcytisine, and 8 core targets were found through network analysis, namely CHRNA3, DRD2, PRKCA, CDK1, CDK2, CHRNA5, MMP1, and MMP9. Additionally, further molecular docking simulation confirmed that CHRNA3, DRD2, PRKCA, CDK1, CDK2, MMP1, and MMP9 had good binding affinities with the corresponding compounds. The GO enrichment analysis showed that the targets of CKI in the treatment of LC might be closely associated with certain biological processes and molecular functions, such as G1/S transition of mitotic cell cycle, positive regulation of ERK1 and ERK2 cascade, and so on. Furthermore, the KEGG pathway enrichment analysis suggested that CKI might simultaneously act on a variety of signal transduction pathways associated with the pathogenesis of LC, including pathways in cancer, proteoglycans in cancer, PI3K-Akt signaling pathway, non-small-cell lung cancer, and small cell lung cancer.

In summary, the current study employed the network pharmacology method to investigate the complex network relationship between multiple components, targets, and pathways for CKI in the treatment of LC. The results validated and predicted the molecular mechanism of CKI in LC at a system level, which might provide insight into the mechanisms of CKI and other anticancer TCMs and facilitate the widespread application of CKI in treating LC. However, the results from our research are based on computational analysis, and further experiments are needed to verify these hypotheses.

## Figures and Tables

**Table 1:** The docking information of 7 targets with corresponding compounds.

Number	Target	PDBID	Compound	CID	Docking score
1	CHRNA3	4ZK4	isomatrine	5271984	6.802
			baptifoline	621307	6.787
			matrine	91466	6.783
			sophoridine	165549	6.731
			lamprolobine	87752	6.123
			N-methylcytisine	670971	6.069

2	DRD2	2HLB	baptifoline	621307	6.616
			sophocarpine	115269	6.486
			N-methylcytisine	670971	6.370

3	PRKCA	4RA4	9*α*-hydroxymatrine	15385684	6.657
			sophoranol	12442899	6.635
			adenine	190	6.058

4	CDK1	5LQF	macrozamin	9576780	6.111
			adenine	190	5.385

5	CDK2	2R3I	macrozamin	9576780	5.933
			adenine	190	5.712

6	MMP1	1SU3	adenine	190	5.508
			oxymatrine	114850	5.371

7	MMP9	1ITV	adenine	190	6.068
			matrine	91466	5.464

## Data Availability

The data used to support the findings of this study are available from the corresponding author upon request.

## Supplementary Materials

Supplementary Materials Supplementary materials contain four tables. Supplementary Table S1: information about the 16 compounds of Compound Kushen Injection. Supplementary Table S2: 97 targets related to LC. Supplementary Table S3: the information of 26 nodes in the PPI network of LC targets. Supplementary Table S4: 27 potential targets of CKI for treating LC.
