# Supplementary material for: Mechanisms of Compound Kushen Injection for the Treatment of Lung Cancer Based on Network Pharmacology
Source: Evid Based Complement Alternat Med. 2019 May 28;2019:4637839. doi: 10.1155/2019/4637839 (PMC6558614; doi:10.1155/2019/4637839)
Supplement: Supplementary Materials — contain four tables. Supplementary Table S1: information about the 16 compounds of Compound Kushen Injection. Supplementary Table S2: 97 targets related to LC. Supplementary Table S3: the information of 26 nodes in the PPI network of LC targets. Supplementary Table S4: 27 potential targets of CKI for treating LC. [file 4637839.f1.zip › 4637839.f1/Table S1.docx]

Table S1. Information about the 16 compounds of CKI.

| **Compound** | **Pubchem CID** | **MW (g/mol)** | **Structure** |
| --- | --- | --- | --- |
| 9α-hydroxymatrine | 15385684 | 264.369 | 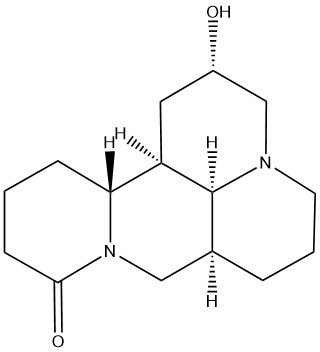 |
| adenine | 190 | 135.13 | 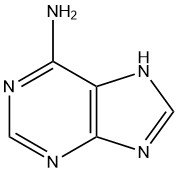 |
| baptifoline | 621307 | 260.337 | 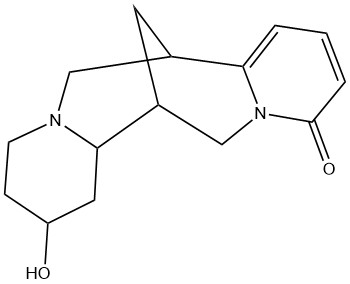 |
| isomatrine | 5271984 | 248.37 | 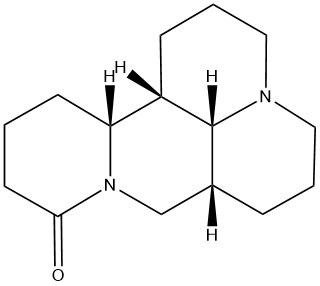 |
| lamprolobine | 87752 | 264.369 | 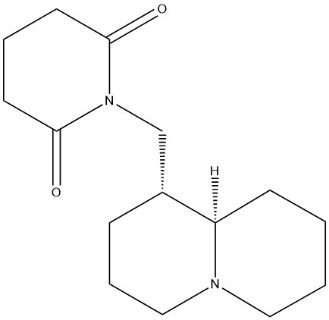 |
| liriodendrin | 21603207 | 742.724 | 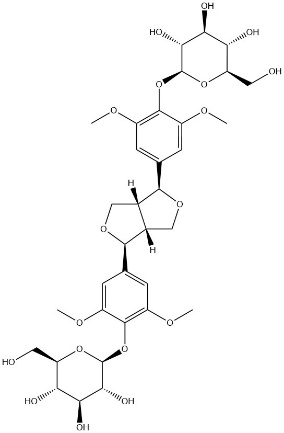 |
| macrozamin | 9576780 | 384.338 | 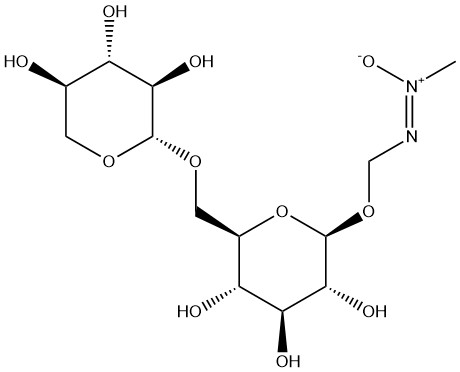 |
| matrine | 91466 | 248.37 | 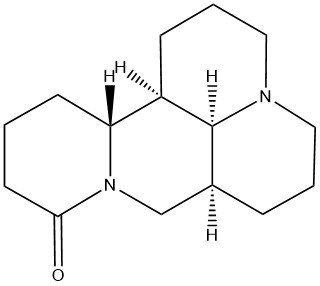 |
| N-methylcytisine | 670971 | 204.273 | 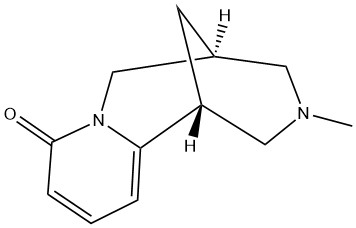 |
| oxymatrine | 114850 | 264.369 | 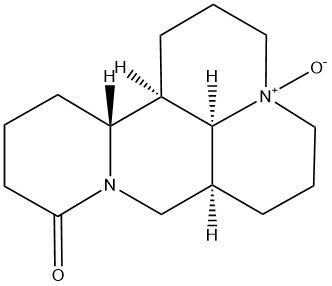 |
| oxysophocarpine | 24721085 | 262.353 | 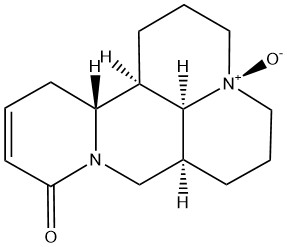 |
| piscidic acid | 6710641 | 256.21 | 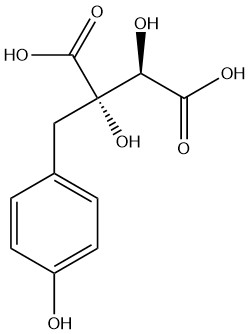 |
| sophocarpine | 115269 | 246.354 | 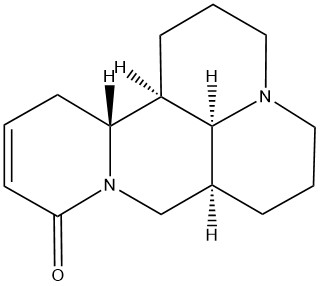 |
| sophoranol | 12442899 | 264.369 | 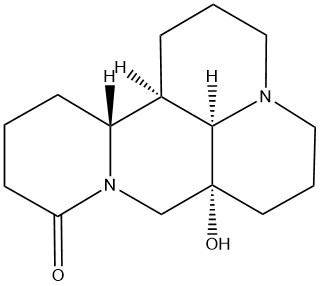 |
| sophoridine | 165549 | 248.37 | 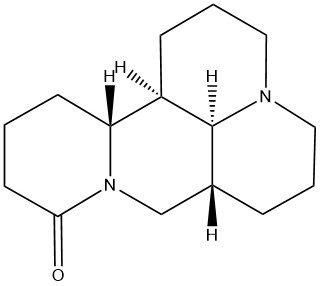 |
| trifolirhizin | 442827 | 446.408 | 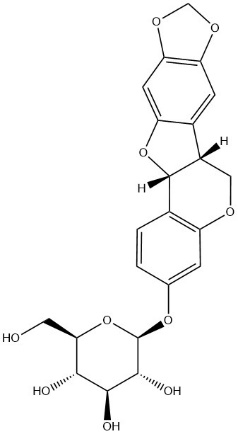 |
